# Ear, nose and throat injuries at Bugando Medical Centre in northwestern Tanzania: a five-year prospective review of 456 cases

**DOI:** 10.1186/1472-6815-13-4

**Published:** 2013-03-25

**Authors:** Japhet M Gilyoma, Phillipo L Chalya

**Affiliations:** 1 Otorhinolaryngology unit, Bugando Medical Centre, Mwanza, Tanzania; 2 Department of Surgery, Catholic University of Health and Allied Sciences, Mwanza, Tanzania

**Keywords:** ENT injuries, Causes, Injury patterns, Outcome, Tanzania

## Abstract

**Background:**

Injuries to the ear, nose and throat (ENT) regions are not uncommon in clinical practice and constitute a significant cause of morbidity and mortality in our setting. There is dearth of literature on this subject in our environment. This study was conducted to describe the causes, injury pattern and outcome of these injuries in our setting and proffer possible preventive measures.

**Methods:**

This was a descriptive prospective study of patients with ear, nose and throat injuries managed at Bugando Medical Centre between May 2007 and April 2012. Ethical approval to conduct the study was sought from relevant authorities. Statistical data analysis was performed using SPSS computer software version 17.0.

**Results:**

A total of 456 patients were studied. The median age of patients at presentation was 18 years (range 1 to 72 years). The male to female ratio was 2:1. The commonest cause of injury was foreign bodies (61.8%) followed by road traffic accidents (22.4%). The ear was the most common body region injured accounting for 59.0% of cases. The majority of patients (324, 71.1%) were treated as an outpatient and only 132(28.9%) patients required admission to the ENT wards after definitive treatment. Foreign body removal and surgical wound debridement were the most common treatment modalities performed in 61.9% and 16.2% of cases respectively. Complication rate was 14.9%. Suppurative otitis media (30.9%) was the commonest complication in the ear while traumatic epistaxis (26.5%) and hoarseness of voice (11.8%) in the aero-digestive tract were commonest in the nose and throat. The overall median length of hospital stay for in-patients was 8 days (range 1 to 22 days). Patients who developed complications and those who had associated injuries stayed longer in the hospital (P < 0.001).

Mortality rate related to isolated ENT injuries was 1.3% (6 deaths). The majority of patients (96.9%) were treated successfully and only 3.1% of cases were discharged with permanent disabilities.

**Conclusion:**

Injuries to the ENT regions are not uncommon in our environment and foreign bodies constitute a significant cause of injury. Majority of these injuries can be prevented through public enlightenment campaigns.

## Background

Injuries to the ear, nose and throat (ENT) regions are not uncommon in clinical practice and constitute a significant cause of morbidity and mortality resulting from increased costs of care and varying degrees of physical, functional and cosmetic disfigurement [1,2]. Studies have shown that ENT injuries are avoidable cause of death and disability [3-5]. In Bugando Medical Centre, ENT injuries are a single most common cause of ENT admissions and contribute significantly to high morbidity and mortality [6,7].

The causes and mechanism of ENT injuries have been reported to vary with age and geographic distribution [3,4,7]. ENT injuries occur in all age groups; however the mechanisms and causes differ between children and adults [3,4,8]. Injuries such as foreign body in the ear, nose and throat remain the commonest and tend to occur more in children with serious complications [9].

In adults, the common etiologies of ENT injuries, across the world, are road traffic accidents, assaults, falls and sports [4]. The types of injury in the sub-Saharan region are different from those in the developed countries [8]. Road traffic accident is reported to be the leading cause of ENT injuries in developing countries, while interpersonal violence is the leading cause in developed countries [4]. Injuries to the ear, nose and throat can occur as an isolated injury or may be associated with multiple injuries to the head, chest, abdominal, spinal and extremities [1,9].

ENT injuries have various mechanisms of injury, blunt traumas such as blows and slaps to the ears represent a different spectrum of injuries [10]. Domestic violence and abuse from law enforcement agents are implicated causes of traumatic tympanic membrane perforation [10].

Most tympanic perforations, besides, penetrating and open injuries to the throat are often life threatening with more dramatic presentations [11]. The less dramatic the injury, the longer it takes for the patient to present to a health facility. However, trauma to the nose with epistaxis or foreign body in the esophagus and airway tend to present as emergencies because of either airway obstruction or dysphagia [8,12].

While foreign bodies in the ear and nose can be easily removed under vision in the clinic [13], those in the throat often present as emergency and are removed in the theatre [12]. However, in developing countries, patients with foreign bodies in the ears and nose often present late after having been attempted by an unskilled health worker and this may often end up with complications which require hospitalization. More often than not this poses more challenges to the otolaryngologist practicing in these countries [13].

In the developing countries like Tanzania, the morbidity and mortality associated with ENT injuries remain a significant but neglected problem. Little work has been done on this subject in our local environment despite increase in the number of admissions of this condition. It is on this background that this study, seeks to examine the causes, pattern and outcome of injuries to the ear, nose and throat regions as seen in our institution and to have a baseline for future comparison.

## Methods

### Study area and design

This was a descriptive prospective study of patients with ENT injuries that were managed in the ENT/Surgery department of Bugando Medical Centre (BMC) from May 2007 to April 2012. BMC is a consultant, tertiary care and teaching hospital for the Catholic University of Health and Allied Sciences-Bugando (CUHAS-Bugando) and has 1000 beds. BMC is one of the four largest referral hospitals in the country and serves as a referral centre for tertiary specialist care for a catchment population of approximately 13 million people from all regions in the northwestern Tanzania. Department of Ear, Nose and Throat is one of the hospital departments with services, covering the above mentioned region in terms of patients’ coverage. There is no trauma centre or established advanced pre-hospital care in Mwanza city as a result all trauma patients are referred to BMC for expertise management.

### Study population

Subjects for the study included all patients of ENT injuries of all age groups and gender irrespective of injury severity who was managed at BMC during the study period and who consented for the study. Patients who died before initial assessment and unconscious patients who had no relative to consent for the study on their behalf were excluded from the study. Recruitment of patients to participate in the study was done at the A & E department. Patients were screened for inclusion criteria and those who met the inclusion criteria were, after informed consent to participate in the study, consecutively enrolled into the study.

All study patients were first resuscitated in the A & E department according to Advanced Trauma Life Support (ATLS). Patients with minor injuries and those with foreign bodies in the ears and nose were treated as outpatients were treated as an outpatient and only patients with moderate to severe injuries and those with foreign bodies in the throat and associated injuries required admission to the ENT wards after definitive treatment in theatre. The severity of injury was determined using the Kampala trauma score II (KTS II) [14]. Severe injury consisted of a KTS II ≤ 6, moderate injury 7–8, and mild injury 9–10.

Depending on the type of injury, the patients were treated either conservatively or by surgery. All patients were followed up till discharged or death. This information was collected using a pre-tested questionnaire. Included in the questionnaire were socio-demographic data, mechanism of injury, pre-hospital care, injury-arrival interval, type and pattern of injury, trauma scores (KTS II), body region injured (ENT), presence or absence of associated injuries, treatment offered, complications of treatment. Outcome variables were length of hospital stay, mortality and disability. The term disability was defined according to the World Health Organization as *any restriction or lack (resulting from any impairment) of ability to perform an activity in the manner or within the range considered normal for a human being”.*

### Statistical data analysis

Statistical data analysis was done using SPSS software (Statistical Package for the Social Sciences, version 17.0, SPSS Inc, Chicago, Ill, USA). Data was summarized in form of proportions and frequent tables for categorical variables. Continuous variables were summarized using range and median. P-values were computed for categorical variables using Chi-square (χ2) test and Fisher’s exact test depending on the size of the data set. Independent student *t*-test was used for continuous variables. Multivariate logistic regression analysis was used to determine predictor variables that are associated with outcome. A p-value of less than 0.05 was considered to constitute a statistically significant difference.

### Ethical considerations

The study was carried out after the approval by the department of surgery and BMC/CUHAS-Bugando ethics review board. An informed written consent was sought from patients or relatives.

## Results

### Demographic profile

During the study period, a total of 530 patients with ENT injuries were managed at Bugando Medical Centre. Of these, 456 patients met the inclusion criteria and these were analyzed. The age of patients at presentation ranged from 1 year to 72 years with a median age of 18 years. The modal age incidence was 1–10 years accounting for 202 (44.3%) patients. There were 302 (66.2%) males and 154 (33.8%) females with a male to female ratio of 2: 1. The majority of patients, 321 (70.4%) came from the urban areas located in Mwanza City.

### Circumstances of injury

Regarding the time of injury, 351 (77.0%) patients sustained injury during the day, 55 (12.1%) at night and in 50 (10.9%), the time was not specified. The commonest cause of injury was foreign bodies (61.8%) followed by road traffic accidents (22.4%) (Table 1). Injuries from foreign bodies in the present were found to be commonest in the pediatric population. Most of injuries occurred at home (288, 63.2%) and on the road (102, 22.4%). This was followed by school and recreation in 20(4.4%) and 14(3.7%) patients respectively. The place of injury was not documented in 32 (7.0%) patients. The majority of injuries, 429(94.1%) were unintentional and the remaining 27(5.9%) injuries were intentional mainly due to assault and suicidal attempt.

**Table 1:** Distribution of the cause of injury versus site of injury

**Cause of injury/site of injury**	**Ear (N/%)**	**Nose (N/%)**	**Throat (N/%)**	**Total (N/%)**
Foreign bodies	160(35.1)	40(8.8)	82(18.0)	282 (61.9)
Road traffic accidents	68(14.9)	34(7.5)	**-**	102(22.4)
Falls	20(4.4)	5(1.1)	**-**	25(5.5)
Assault	13(2.9)	5(1.1)	2(0.4)	20(4.4)
Burns	5(1.1)	5(1.1)	**-**	10(2.2
Animal bite	3(0.7)	7(1.5)	**-**	10(2.2)
Iatrogenic	**-**	2(0.4)	5(1.1)	7(1.5)
Total	**269(59.0)**	**98(21.5)**	**89(19.5)**	**456(100)**

There were no cases of indeterminate intent. In this study, only 23 (5.7%) patients received pre-hospital care. The vast majority of patients, 368 (80.7%) reported to the A & E department late (i.e. more than 24 hours after injury) with only 88(19.3%) presenting within 24 hours of injury. The injury-arrival time, defined as the time interval taken from injury and reception at the A& E Department ranged from 1 hour to 8 days with a mean of 18 hours. The median waiting time (i.e. time interval taken from reception at the A& E Department and reception of treatment) was 4 hours (range 1–6 h). The majority of patients, 404(88.6%) were attended to within 1–4 hours of arrival to the A & E department. The remaining 52 (11.4%) patients had delayed definitive treatment.

### Injury characteristics/clinical presentations

In this study, foreign body insertion and blunt injuries were the most frequent mechanism of injury in all sites (ENT) and accounted for 382 (83.8%) patients. The remaining 74 (16.2%) patients had either penetrating or combined (blunt and penetrating) injuries. The ear was the most common body region injured accounting for 59.0% of cases. In the ear, foreign body insertion and blunt trauma such as blows and slaps was the highest form of injury. The nose and throat injuries were recorded in 21.5% and 19.5% of patients respectively (Table 1). Coins were the most common type of foreign body in the throat occurring in 54 (65.9%) patients, whereas groundnuts and beans were the most common type of foreign body in the ear and the nose in 140 (87.5%) and 38 (70.0%) patients respectively. Isolated ENT injuries were reported in 402(88.2%) patients while 54 (11.8%) patients had multiple injuries. Associated injuries were reported in 54(11.8%) patients. Of these, head and musculoskeletal injuries were the most common associated injuries accounting for 30 (55.6%) and 10 (18.1%) respectively. Other associated injuries included chest, abdominal and spinal injuries. Generally, according to Kampala trauma score II (KTS II), the majority of patients (328, 71.9%) had mild injuries and the remaining 128 (28.1%) patients had moderate to severe injuries. As shown in Table 2, foreign body insertion/ingestion was the most frequent clinical presentation of patients.

**Table 2:** Distribution of patients according to clinical presentation

**Anatomical site**	**Clinical presentation**	**Frequency**	**Percentage**
Ear	Foreign body insertion	160	35.1
	Hearing loss	34	7.5
	Otalgia	30	6.6
	Mucoid otorrheoa	30	6.6
	Tinnitus	24	5.3
	Lacerations/cuts of the pinna	20	4.4
	Bleeding	17	3.7
	Burns	5	1.1
Nose	Foreign body insertion	40	8.8
	Traumatic epistaxis	24	5.3
	Rhinorrhoea	18	3.9
	Lacerations/cuts	17	3.7
	Anosmia	5	1.1
	Burns	5	1.1
	Nasal obstruction	4	0.9
Throat	Foreign body ingestion/aspiration	82	18.0
	Odynophagia	23	5.0
	Open neck wounds	2	0.4
	Aphonia	2	0.4

### Admission pattern and treatment modalities

The majority of patients (324, 71.1%) were treated as an outpatient and the remaining 132(28.9%) patients required admission to the ENT wards after definitive treatment. Most of patients with foreign bodies in the ear and nose and those with minor ENT injuries were treated as outpatients. Associated injuries were treated accordingly. Foreign body removal and surgical wound debridement were the most common treatment modalities performed in 61.9% and 16.2% of cases respectively. Table 3 shows treatment modalities for both isolated and associated injuries.

**Table 3:** Treatment modalities for both isolated ENT and associated injuries

**Treatment modality**	**Frequency**	**Percentage**
Foreign body removal	282	61.9
Surgical wound debridement/wound dressing	74	16.2
Adrenaline nasal packs/epistaxis catheters	10	2.2
Plastic surgery of the pinna (pinnaplasty)	10	2.2
Nasal deformity reconstruction (rhinoplasty)	9	2.0
Treatment of fractures	9	2.0
Exploratory laparotomy	4	0.9
Craniotomy ± barr holes	3	0.7
Tracheostomy	2	0.4

### Outcome and follow up of patients

Complications related to isolated ENT injuries were recorded in 68(14.9%) patients. Generally, the ear had the highest number of complications (86.8%), of which suppurative chronic otitis media was the most common complication accounting for 30.9% of cases (Table 4).

**Table 4:** Complications associated with ENT injuries (N = 68)

**Anatomical site**	**Complications**	**Frequency**	**Percentage**
**Ear**		**(59)**	**(86.8)**
	Chronic otitis media	21	30.9
	Sensorineural hearing loss	16	23.5
	Loss pinna	12	17.6
	Perforated tympanic membrane	6	8.8
	Facial palsy	4	5.9
**Nose**		**(31)**	**(45.6)**
	Traumatic epistaxis	18	26.5
	Nasal deformity	8	11.8
	Septal abscess	5	7.4
**Throat**		**(9)**	**(13.3)**
	Hoarseness of voice	8	11.8
	Laryngeal stenosis	1	1.5

The overall length of hospital stay for in-patients ranged from 1 to 22 days with a median of 8 days. Patients who developed complications and those who had associated injuries stayed longer in the hospital (*p* < 0.001).

In this study, thirty-one patients died giving an overall mortality rate of 6.8%. Mortality rate was higher in patients with ENT injuries associated with multiple injuries (25 deaths, 80.6%) than in those with isolated ENT injuries (6 deaths, 19.4%). This difference was statistically significant (*p* = 0.011). Generally, the overall mortality rate related to isolated ENT injuries was 1.3% (6 deaths).

The follow up periods ranged between 3–6 months as the case warranted. Out of 425 survivors, 412 (96.9%) were discharged well and the remaining 13 (3.1%) were discharged with permanent disabilities related to loss of pinna and permanent tracheostomy due to traumatic laryngeal stenosis.

## Discussion

Injuries to the ear, nose and throat regions are a common but neglected form of trauma and constitute a significant cause of morbidity and mortality worldwide [1,2,7]. In this review, ENT injuries were found to be most common in the first decade of life and tended to affect more males than females. Similar demographic observation was also reported by other authors [1,5].

This could be explained by the fact that these were the active and assertive age group that can be involved in high risk activities such as insertion/ingestion of foreign bodies, fights, climbing or jumping from heights.

In our study, males were more affected than females with a male to female ratio of 2:1 which is in agreement with other studies [1,5,7,8,12]. The reasons for the male preponderance in our series may be attributed to the overactive nature of males as compared to their female counterparts.

Most of the patients in the present study came from the urban areas located in Mwanza City. Similar observation was also reported by Aremu *et al.*[1], but at variant with Singh *et al.*[2] who reported that most of patients were from rural areas. The reason for high number of patients from urban areas in our study may be attributed to the fact that our hospital is located in the urban area in Mwanza city.

The commonest cause of injury in our study was foreign bodies followed by road traffic accidents. Injuries from foreign bodies in the present were found to be commonest in the pediatric population. Similar etiological pattern of ENT injuries was also reported by others [3,5,12], but in contrast to other authors [8,15] who reported fall and trauma during playing to be the commonest mode of injuries.

More than sixty percent of injuries in our series occurred at home which is in agreement with other studies [7,8,12]. The home remains a dangerous place especially for children as lack of enough supervision may result in ENT injures such as ingestion/insertion of foreign bodies and other injuries. High percentage of home occurrence ENT injuries in our study reflects lack of coordination and unawareness of dangerous substances especially in children, poor supervision and lack of domestic safety measures.

The pre-hospital care of trauma patient has been reported to be the most important factor in determining the ultimate outcome after the injury [16,17]. In this study, only 5.7% patients received pre-hospital care. Similar observations have been noted in other studies in developing countries [7,8]. The lack of advanced pre-hospital care in our environment coupled with ineffective ambulance system for transportation of patients to hospitals are a major challenges in providing care for trauma patients including ENT trauma.

More than eighty percent of patients reported to the A & E department later than 24 hours after injury which is in keeping with other study done elsewhere [3,8]. Late presentation in the present study may be attributed to delay in referral from private and public clinics, dispensaries and health centers, self-treatment at home, consultation with traditional healers and transport costs. Delayed presentation following trauma increases the likelihood of death, complications as well as prolonged hospital stay.

In agreement with other studies [3,5], the ear was the most common body region injured. This is at variant with Arif & Saatea [8] who reported nasal trauma as the commonest type of injury. In the ear, foreign body insertion was the highest form of injury which is in contrast with Matilda in Nigeria [18] who reported blows and slaps as the most common cause of otologic injuries.

The clinical presentations depend on the region involved. In this study, clinical presentations did not differ significantly from other studies [3,5]. The presentation was more dramatic in the airway with presentation of dyspnea and stridor. However, patients with foreign bodies in the throat were less dramatic, presenting with dysphagia, odynophagia as the common features.

The presence of associated injuries is an important determinant of the outcome of injuries including ENT patients [15]. ENT injuries are commonly associated with other injuries and these may complicate the management and affect the outcome [1,9]. In the present study, the presence of associated injuries was found to be significantly associated with both mortality and length of hospital stay (morbidity). Early recognition and treatment of associated injuries is important in order to reduce mortality and morbidity associated with ENI injuries.

In this study, more than 70% of patients with foreign bodies in the ear and nose and those with minor ENT injuries were treated as outpatients were treated as an outpatient and only 28.9% of patients required admission to the ENT wards after definitive treatment. Foreign body removal and surgical wound debridement were the most common treatment modalities performed. All the foreign bodies in the ear and nose were successfully removed, that is 100% treatment success rate. This treatment pattern was also reported elsewhere by others [3,5,8]. The reasons for the low rate of ENT admissions in this study may be attributed to the fact that the majority of our patients presented with minor injuries that did not require admission following definitive treatment. Only patients with moderate to severe injuries and those with associated and multiple injuries were admitted.

The presence of complications has an impact on the final outcome of patients presenting with ENT injuries as supported by the present study. The pattern of complications in the present study is similar to what was reported by others [3,8,9]. In our study, suppurative otitis media was the commonest complication in the ear while traumatic epistaxis and hoarseness of voice were the commonest complications in the nose and throat respectively. Of interest, some of complications in the ear and nose resulted from foreign bodies that have been tampered with by the unqualified health personnel and ended up with perforated tympanic membrane and traumatic epistaxis. This observation is more common in most centres in developing countries such as Tanzania and poses more challenges to the otolaryngologist in these countries [7]. The high number of patients with suppurative otitis media following traumatic tympanic membrane perforation was due to the common habit of instilling ear drops into the external auditory canals and meticulous cleaning of blood from the external auditory canal following these injuries. These habits delay the healing of the tympanic membrane thus making the middle ear more prone to infections [3,13]. Early recognition and management of complications following ENT injuries is of paramount in reducing the morbidity and mortality resulting from these injuries.

The length of hospital stay (LOS) has been reported to be an important measure of morbidity among trauma patients. Prolonged hospitalization is associated with an unacceptable burden on resources for health and undermines the productive capacity of the population through time lost during hospitalization and disability [19]. The overall median LOS in this study was relatively higher compared to that reported in Nigeria [1]. Patients who developed complications and those who had associated injuries in our study contributed significantly to prolonged LOS.

The overall mortality rate related to isolated ENT injuries in the present study was 1.3%, a figure which is relatively high compared with Matilda *et al.*[3] who reported no mortality in their series. This low mortality rate in our study may be attributed to the large number of patients with mild injuries.

Generally, the outcome of patients in this study was satisfactory as more than 95 percent of patients were treated successively and discharge well with no permanent disabilities. Late presentation and exclusion of large number of patients from the study were the major limitation in this study. However, despite these limitations, the study has provided local data that can be utilized by health care providers to plan for preventive strategies as well as establishment of management guidelines for patients with ENT injuries.

## Conclusion

Injuries to the ear, nose and throat constitute a major cause of ENT admissions in this environment with foreign body as the commonest cause of injury. The young adults that represent the workforce are the population mainly affected. Most of patients in our local setting present late with increased risk of complications. Majority of these injuries can be prevented through public enlightenment campaigns. Early recognition and treatment of ENT injuries is important in order to reduce mortality and morbidity associated with these injuries.

## Competing interests

The authors declare that they have no competing interests.

## Authors’ contributions

JMG conceived the study and did the literature search, coordinated the write-up, editing. PLC participated in the literature search, writing of the manuscript, editing and submission of the article. All the authors read and approved the final manuscript.

## Pre-publication history

The pre-publication history for this paper can be accessed here:

http://www.biomedcentral.com/1472-6815/13/4/prepub

## References

[B1] Aremu SK Alabi BS Segun-Busari SW Omotoso SW Audit of Pediatric ENT Injuries Int J Biomed Sci 2011 7 218 221 23675239 PMC3614839

[B2] Singh I Gathwala G Gathwala L Yadav SPS Wig U Ear, Nose and Throat injuries in children Pak J Otolaryngol 1993 9 133 135

[B3] Matilda I Lucky O Chibuike N Ear, nose and throat injuries in a tertiary institution in Niger delta region Nigeria J Med Res Prac 2012 1 59 62

[B4] Arif RK Naseem U Inayat U Shah ED Noor SK Causes and complications of ear, nose and throat injuries in children. A study of 80 cases J Med Sc 2006 14 1 57 59

[B5] Sogebi OA Olaosun AO Tobih JE Adedeji TO Adebola SO Pattern of ear, nose and throat injuries in children at Ladoke Akintola University of technology teaching hospital, Osogbo, Nigeria Afric J 2006 3 61 63

[B6] Bugando Medical Centre (BMC) Medical record database, 2010/2011

[B7] Gilyoma JM Chalya PL Endoscopic procedures for removal of foreign bodies of the aerodigestive tract: The Bugando Medical Centre experience BMC Ear, Nose Throat Disorders 2011 11 2 10.1186/1472-6815-11-2 PMC3033844 21255409

[B8] Arif RK Saatea A Ear, nose and throat injuries in children Ayub med Coll Abbottabad 2005 17 54 56 15929529

[B9] Figueriedo RR Azevedo AA Kos AO Tomita S Complications of Ear, nose and throat foreign bodies Braz J Otorhinolaryngol 2008 74 7 15 18392495 10.1016/S1808-8694(15)30744-8 PMC9450582

[B10] Orji FT Non-explosive blast injury of the tympanic membrane in Umuahia, Nigeria Nig J Med 2009 18 365 369 10.4314/njm.v18i4.51227 20120138

[B11] Okoye BC Oteri AJ Cut throat injuries in Port Harcourt Sahel Medical J 2001 4 207 209

[B12] Endican S Garap JP Dubey SP Ear, nose and throat foreign body in Melanesian children: an analysis of 1037 cases Int J Pediatr Otorhinolaryngol 2006 70 1539 1545 10.1016/j.ijporl.2006.03.018 16707167

[B13] Okoye BC Onotai LO Foreign body in the nose Niger J Med 2006 15 301 304 17111765 10.4314/njm.v15i3.37235

[B14] Mutooro SM Mutakooha E Kyamanywa P A comparison of Kampala trauma score II with the new injury severity score in Mbarara University Teaching Hospital in Uganda East Cent Afr J Surg 2010 15 62 70

[B15] Synders LC Jian VN Saltzman DA Strate RG Perry JE Leonard AS Blunt Trauma in Adults and Children, a comparative analysis J Trauma 1990 30 1239 1245 10.1097/00005373-199010000-00008 2213932

[B16] Trunkey Donald D Maull Kimball I Trunkey Donald D, Lewis Frank R Prehospital Trauma care Current Therapy of Trauma 1999 4 Philadelphia: Mosby 121 122

[B17] Liberman M Mulder D Lavoie A Denis R Sampalis JS Multicenter Canadian study of prehospital trauma care Ann Surg 2003 237 2 153 160 12560770 10.1097/01.SLA.0000048374.46952.10 PMC1522139

[B18] Matilda I Otologic injuries in Port Harcourt J Med Med Sci 2012 3 745 748

[B19] Kang EG Sharma GK Lozano R The global burden of injuries Am J Public Health 2000 90 523 526 10754963 10.2105/ajph.90.4.523 PMC1446200

